# An assessment of monitoring requirements and costs of 'Reduced Emissions from Deforestation and Degradation'

**DOI:** 10.1186/1750-0680-4-7

**Published:** 2009-08-26

**Authors:** Hannes Böttcher, Katja Eisbrenner, Steffen Fritz, Georg Kindermann, Florian Kraxner, Ian McCallum, Michael Obersteiner

**Affiliations:** 1 International Institute for Applied Systems Analysis, Schlossplatz 1, A-2361 Laxenburg, Austria; 2 Ecofys GmbH, Am Wassermann 36, 50829 Cologne, Germany

## Abstract

**Background:**

Negotiations on a future climate policy framework addressing Reduced Emissions from Deforestation and Degradation (REDD) are ongoing. Regardless of how such a framework will be designed, many technical solutions of estimating forest cover and forest carbon stock change exist to support policy in monitoring and accounting. These technologies typically combine remotely sensed data with ground-based inventories. In this article we assess the costs of monitoring REDD based on available technologies and requirements associated with key elements of REDD policy.

**Results:**

We find that the design of a REDD policy framework (and specifically its rules) can have a significant impact on monitoring costs. Costs may vary from 0.5 to 550 US$ per square kilometre depending on the required precision of carbon stock and area change detection. Moreover, they follow economies of scale, i.e. single country or project solutions will face relatively higher monitoring costs.

**Conclusion:**

Although monitoring costs are relatively small compared to other cost items within a REDD system, they should be shared not only among countries but also among sectors, because an integrated monitoring system would have multiple benefits for non-REDD management. Overcoming initialization costs and unequal access to monitoring technologies is crucial for implementation of an integrated monitoring system, and demands for international cooperation.

## Background

Globally, by far, the biggest greenhouse gas mitigation potential in forestry is reducing emissions from deforestation. The negotiations on a future REDD (Reduced Emissions from Deforestation and Degradation) policy framework are ongoing and many options exist for its implementation [1]. REDD activities will need to be based on scientifically robust estimates of emissions if they are to be effective. This requires methodologies for Monitoring, Reporting and Verification (MRV) of emissions that follow the United Nations Framework Convention on Climate Change (UNFCCC) principles of transparency, consistency, comparability, completeness, and accuracy [2]. Practicable approaches for monitoring changes in forest and vegetation carbon for REDD will involve the interpretation of remotely sensed imagery (including both airborne and satellite imagery). For many potential REDD applications, remote sensing technologies for REDD are often no longer technically constrained, as has been shown by several studies for many regions [3-5]. A variety of methods can be applied depending on national capabilities, available resources, deforestation patterns and forest characteristics. Porrùra et al. [6] as well as Achard et al. [5] identified the following key requirements for implementing national systems for monitoring REDD: international commitment of resources to increase capacity, coordination of observations, standardized consensus protocols, and access to data at the appropriate resolution at low costs.

A key element of the REDD discussion are monitoring costs. Their estimation and extent will have an impact on the success of REDD mechanisms. Monitoring costs, however, will depend also on the scope and implementation of REDD mechanisms. Elements that will have an influence on the costs include the payment scheme for REDD, whether it is market or non-market based or a combination of such. The scope of the system, either national or sub-national, will have an impact on the costs as well as the type and level of verifications that will be applied. In this article we identify key elements of REDD policy, evaluate requirements for monitoring efforts and assess their costs.

## Results

### Evaluation of monitoring requirements associated with key elements of REDD policies

REDD policies and REDD monitoring systems will co-evolve. A REDD monitoring system needs to be designed to serve known current and future REDD policy requirements conditional on technical capabilities and costs. Likewise, future REDD policy designs will need to be based on comprehensive, international consistent and accurate spatially explicit data on global forest change and carbon stocks, emissions and trends. Herold and Johns, [7] define a REDD monitoring framework with a set of "minimum common characteristics" to provide a starting point for actors to engage in implementation activities, and to support REDD early actions and readiness mechanisms for building national REDD monitoring systems. Here we define a list of elements extracted from the recently proposed REDD policy approaches that are translated into monitoring requirements.

Under the UNFCCC and Kyoto Protocol, no climate policies exist to reduce emissions from deforestation or forest degradation in developing countries. In December 2005, COP-11 established a two-year process to review relevant scientific, technical, and methodological issues and to consider possible policy approaches and positive incentives for reducing emissions from deforestation in developing countries [8,9]. Recent research suggests a broad range of possible approaches to effectively reduce emissions from tropical deforestation and forest degradation, e.g.; [10-12].

Most approaches suggest voluntary participation and require an assessment of historic and future deforestation rates based on detectable change in forest area using remote sensing imagery. The use of positive incentives as a source of finance for activities and policies is the ultimate basis of all proposals. The scope and design of the REDD approach (to be finally adopted) has implications for monitoring and verification efforts. From the proposals analyzed in this study we identified several aspects relevant for monitoring (in general) and for costs of remote sensing, change detection and verification (in particular) (see Table 1).

**Table 1:** Main elements of different proposals for approaches to reduced deforestation and degradation (based on [58,59]).

**Element**	**Examples of variation in implementation**
Definition of forests, deforestation and degradation	National definitions Technically detailed versus general (e.g. three classes: forest, degraded forest, non-forest as proposed by Joint Research Centre) Marrakech accords (UNFCCC 2002): 0.05–1 ha minimum area, 10–30% tree canopy cover and a potential of 2–5 m tree height; used by Annex I country Kyoto reporting and CDM projects Others see Mollicone et al. [16] (intact forest, non-intact forest, non-forest) Deforestation versus deforestation **and **degradation

Scale	National versus projects
Minimum Mapping Unit	National, sectoral
Target area	Sub-national
	Projects
	Definition of MMU

Reference level, baseline	National historical averages with a correction for countries which have already significantly reduced deforestation; compared to reference (e.g. 1990 or 2000)
Data for baseline	
Baseline development	
	Global average deforestation rate, countries with less than half the global average will be credited for not increasing deforestation, geographical
	Sophisticated prognostic **model **of land competition

Carbon model	Simple, national average carbon stock versus sophisticated assessment
	Inventory versus IPCC default values
	Simple, national average carbon stock for both
	intact and
	non-intact (degraded) forest
	Detailed carbon maps based on RS

Financing mechanism and trading	Instruments:
	Market-based
	Tax
	Incentives
	Units created for trade: Certified emission reductions (CERs) in CDM projects:
	Short-term credits (tCERs)
	Long-term credits (lCERs)
	Voluntary carbon market:
	Not entire forest area accounted for
	Only specified amount banked as buffer.

Different monitoring options are available to detect change of forest cover and of carbon stocks within forests (see Section "Assessment of monitoring technologies and costs" below). It has to be considered that the decision of which monitoring system will be applied in the final REDD system will not depend on availability of technologies alone. Policy makers will base their decision on the REDD design considering other factors including: definitions, scale and scope of activities, financing mechanisms, trading of credits and their own country context. In the following text we provide an outline of the role of monitoring within a REDD system.

### Definition of forests, deforestation and degradation

According to the Food and Agricultural Organization (FAO), the definition of deforestation refers to a change in land cover with depletion of tree crown cover to less than 10 percent [13]. The UNFCCC defines deforestation as "the direct human-induced conversion of forest land to non-forest land" (paragraph 1(b) of the Annex to Decision 16/CMP.1).

Changes within the forest class (e.g. from closed to open forest) which negatively affect the stand or site and, in particular, lower the production capacity, are termed forest degradation.

The Good Practice Guidance for Land Use, Land-Use Change and Forestry (GPG LULUCF) of the Intergovernmental Panel on Climate Change (IPCC) as well as the 2006 guidelines for Agriculture, Forestry and Other Land Uses (AFOLU) include definitions that might be used as a basis for definitions in a potential REDD mechanism. The options for definitions of forests and deforestation within future policy frameworks might range from the application of existing national definitions to globally harmonized definitions such as the FAO. They might be based on technically detailed descriptions like those prescribed by the Marrakech Accords (UNFCCC 2002: 0.05–1 ha minimum area, 10–30% tree canopy cover and a potential of 2–5 m tree height) and used by Annex I countries for Kyoto reporting and CDM projects. Existing inventories can provide such technical features of forests in developed countries.

Technical requirements and associated costs between a RED (without degradation) and a REDD (including degradation) monitoring system would turn out to be starkly different. Measuring forest degradation through remote sensing is technically more challenging. In the transition from intact to degraded forest the canopy may still be closed (or closed again), whilst the carbon stocks may be reduced by up to 75% [14]. In addition, it may take place far from access features such as roads and rivers where it is even more difficult to detect. Compared to deforestation, degradation has therefore not been quantified in most countries in the past. Recently, Asner et al. [15] showed for an area of over two million square kilometres in the Brazilian Amazon that at least 76% of all selective harvest practices resulted in high levels of canopy damage and significant amounts of biomass removal.

A more general approach discussed for tropical forests and proposed from a monitoring perspective by the Joint Research Centre [16] forms three classes: forest, degraded forest, non-forest. Regardless of how such a classification will look like in detail (number of classes, parameters included in definition of strata, etc.), stratification of the forest landscape to be monitored is key to lower costs and still maintain a high level of precision and accuracy [17].

Recently, the UNFCCC negotiation enhanced the REDD discussion with the inclusion of sustainable forest management and the conservation and enhancement of forest carbon stocks [18]. Monitoring requirements and costs for "REDD plus" could differ from those that focus only on the original REDD notion. However, methodologically similar technologies are involved. The definitions would have to include relevant activities such as afforestation reforestation, sustainable forest management etc.

### Scale and scope

At the current stage it remains unclear if the REDD mechanism will be applied at the national level or sub-national level, or a combination of both. Likewise, it is unclear whether monitoring will in the end be done on the project, country or even global scale. Following a project approach (compared to national level measures like inventories), simplifies quantification and monitoring efforts because of the clearly defined boundaries for project activities, the relative ease of stratification of the project area, and the choice of carbon pools to measure [6]. At the national level, costs will differ significantly between countries as costs for monitoring activities are related to the size of the country, i.e. the area to be monitored [19].

The Minimum Mapping Unit (MMU) required for effective monitoring directly influences the costs of monitoring. Remote sensing data analysis becomes more difficult and more expensive with smaller MMUs, i.e. more detailed MMUs increase mapping efforts and usually decrease change mapping accuracy [7]. For example, using optical remote sensing, the use of 30 m resolution imagery results in a MMU of ca. 0.1 ha, while data with 5 m resolution allows MMUs of 0.01 ha and smaller (RapidEye pers. comm.).

### Emission displacement

GHG emissions displacement might occur when interventions to reduce emissions in one geographical area (sub-national or national) cause an increase in emissions in another area through the relocation of activities. Monitoring of REDD also needs to address this leakage of emissions which will have direct implications for monitoring costs.

Wall-to-wall coverage (i.e. analysis of satellite data that covers the full spatial extent of the forested area), with high resolution satellite imagery or even with airborne imagery will provide a high level of certainty to estimate land-use change [17]. A globally consistent forest carbon observatory with wall-to-wall mapping would partly address the problem of leakage that is often associated with project level approaches. This analysis is ideal, but often not practical due to large areas and constraints on resources for analysis. An alternative approach to wall-to-wall coverage is sampling. Several approaches have been successfully applied to sample within the total forest area to reduce both costs and the time for analysis [17].

However, to be effective, an assessment of displacement of REDD would require a "land based" reporting approach. Clearly, a global forest carbon observatory would probably also yield the lowest cost solution per MRV-REDD unit by reaping the most of economies of scale. Economies of scope relate to the fact that in a "system of systems" approach, such as GEOSS (Global Earth Observation System of Systems), one observing system creates benefits to another. For example, an Earth Observation (EO) satellite system dedicated to yield and acreage estimation in agriculture could at the same time be used for deforestation monitoring. Thus, the cost per unit carbon from REDD will be decreased by its complementary use for agricultural monitoring.

### Additionality and choice of reference level

REDD policies have to address the difficulty in determining additionality compared to a baseline. Historic baselines use national historical averages as reference and compare them to current rates. However, reliable estimates of historical carbon emissions from deforestation and degradation are de facto not available. Considerable (re-)analysis of recently opened remote sensing archives would be necessary. Similarly, global average deforestation rates are discussed that would require less historic deforestation data at the national level. Models for baseline estimations range from relatively simple extrapolations of past trends in land use to more complex extrapolations of past trends using spatially explicit models of land-use change driven by biophysical and socioeconomic factors [20]. Sophisticated prognostic models of land competition that could be employed to provide forward-looking baselines of deforestation pressures (Obersteiner, M. et al.: Avoiding REDD hot air – an IIASA proposal for generating standardized and globally consistent national reference scenarios that maximize sustainability, submitted) require a lot of data streams to be assimilated in these models. Such data streams range from socio-economic census data, forest ownership and governance data to detailed climatic and land use information derived from EO. The overall costing for the latter baseline establishment scenario is more complex than the observing system. It not only covers the physical monitoring of the forest per se, but the entire planning, monitoring and evaluation phase of REDD implementation and other associated policies. Sensitivity analysis within these more complex economic land use models is required to deliver robust cost estimates of avoided deforestation. Moreover, such models can be used to determine the level of accuracy at which the data has to be collected and so determine the cost-benefit relationship of incremental costs versus the incremental benefits of better EO and more detailed socio-economic data [21].

### Carbon model

There is a great diversity of methods for estimating carbon stocks in forests. Therefore, it is extremely important for the planning of forest carbon observation systems to agree internationally on common methods and standards. Three approaches (tiers) for estimating carbon are proposed by the IPCC LULUCF [19,22]. Tier 1 is based on default assumptions and default values for carbon stocks e.g. for different forest types. In tier 2, country-specific carbon stocks are applied to activity data, disaggregated to appropriate scales. In tier 3, countries use advanced estimation approaches that may involve complex models and highly disaggregated data including detailed maps based on remote sensing as well in-situ measurements. Estimates of carbon provided by the GPG tier 3 approach yield the lowest uncertainties, but involve the highest MRV costs.

The GOFC-GOLD sourcebook reviews in detail the question of which tier should be used. The choice is relevant not only for costs but also for the level of total uncertainty. The error in applying a relatively coarse IPCC Tier 1 approach (as compared to carbon stocks estimated from ground plot measurements from six sites around the world) can range between an overestimation of 33% to an underestimation of 44% [17]. The sourcebook further highlights that despite a constant low uncertainty of 5% for the area change component, the uncertainty of the total final estimate of emissions is governed by the higher uncertainty in the carbon stock data. Therefore, it can be said that if uncertainty cannot be reduced to equal levels for the emission factor, "investment in an unbalanced half is money poorly spent" (page 55 in [17]).

It is currently still unclear, which level of minimum precision and accuracy is required under which REDD implementation scenario. Moreover, no decision is made on the system boundaries, i.e. which carbon pools are going to be included in REDD. While including soil carbon pools will increase uncertainty and costs, an integrated forest sector view including harvested wood products might actually decrease relative uncertainty of carbon stock change estimates (compared to total carbon stocks) [23] due to system integration. Thus, also within a REDD framework, even pools with high uncertainty should rather be included by applying conservative default values [2].

### Implementation mechanisms

REDD aims to encourage permanent forest management to ensure that carbon emissions are not occurring. Permanence is therefore the core element of the whole REDD approach and points to the requirement of temporal consistency of monitoring. At the moment, different financing options are discussed including fund based and market based approaches [1]. While a non market based international REDD fund might not have to rely for its operations on detailed carbon accounting (it could instead, in a first phase, focus on capacity building in the forest sector through technical cooperation programs or fund agricultural intensification programs), a market based approach will require detailed measurement of carbon emissions avoided from REDD activities. On the level of (sub-)national REDD programs, the implementation of a deforestation tax system will require different observational capabilities as a REDD carbon trading system or a REDD subsidy program. Furthermore, the observational requirements will vary, as within a country land tenure is not always clearly defined and secure.

### Trading

In a market based approach, carbon credits are traded on the carbon market and paid by private or sovereign clients. Different trading mechanisms have been discussed including different types of credits (e.g. temporary versus permanent credits). Under the CDM projects relating to afforestation and reforestation (A/R), a system of temporary credits has been implemented which differentiates between short-term credits (tCERs) and long-term credits (lCERs) [24].

Short-term credits are given for existing carbon stocks at the time of verification, which expire after 5 years. Verification takes place again every 5 years until the end of the project period, taking changes in carbon stocks into account by adjusting the number of tCERs issued every 5 years [24]. To determine the changes in carbon stocks every 5 years, frequent monitoring is required. In case of forest loss the carbon credit buyer is liable to cover the lost credits; therefore this option is not very attractive to the credit buyer because the entire liability and risk lies with him.

A different approach to issue permanent carbon credits is currently applied on the voluntary carbon market by VCS (Voluntary Carbon Standard) and Carbon Fix standards [25]. This approach is based on the concept that not all forest area or carbon credits are accounted for, but a specified amount of forest area or carbon credits are placed in a buffer. In case forest loss is identified, the buffer is reduced. If no forest is lost over a specified time period the buffered amount can be retrieved, providing an additional incentive to maintain the forest. The size of the buffer is determined through a risk assessment. In this approach, monitoring is important to identify forest loss in a timely manner thus triggering the reduction of the buffer. The incentive system can also be closely linked to the monitoring system as a tiered approach similar to the one applied in IPCC LULUCF and AFOLU. In this case, tiers represent increasing levels of data requirements and with increased tiers the buffer can be reduced. The increased costs for monitoring would be covered through the additional amount of credits received. Different to the tCERs, the carbon credits in the voluntary market are permanent credits and the liability is with the carbon credit seller, covered through the buffer. From this perspective it is more attractive to the carbon market.

Each of these elements discussed above offers various options for implementation. An international agreement might also leave the final implementation to the member states, prescribing only the range within which countries have to choose their definition (i.e. the forest definition in the Kyoto Protocol implementation as defined in the Marrakech Accords).

The more flexibility such an agreement leaves to each country for implementation, the more options a country has for a cost efficient and locally adapted design of its monitoring systems. Thus, it appears that a clear choice for the right monitoring system for the ultimate REDD carbon trading is not yet possible. However, the exchange rate between a REDD unit and an Assigned Amount Unit will surely also depend on the level of measurement uncertainty of individual REDD units. The less MRV a REDD unit will appear, the more it will be discounted.

### Assessment of monitoring technologies and costs

Remote sensing will be an essential method to establish baselines and monitor progress in reducing emissions from deforestation and there will be considerable need to build capacity in this regard in many non-Annex I countries [26,27]. The following section will briefly describe the technologies, with a special focus on their costs.

### Forest area and carbon stock change detection

The assessment of emissions from deforestation and degradation requires data on both change in forest cover and estimates of carbon stock changes associated with transition between land use types [8]. It is an estimation process that includes measured data and the application of models at many levels, with different uncertainties. The IPCC has compiled methods and good practice guidance [22] to move from two-dimensional (forest area) to three-dimensional (carbon stocks) evaluation of changes. It is worth mentioning here, that the IPCC suggests the use of remote sensing technologies only to assess forest area changes, while there are no suggestions for the use for direct biomass estimates. The methodology needs to be consistent at repeated intervals, and results need verification with ground-based or very high resolution remote observations [28]. As Goetz et al. [29] concluded from a review of different approaches to estimate above ground biomass, mapping attempts without satellite imagery are often insufficient while direct remote sensing approaches provided more coherent maps of forest biomass compared to other approaches.

Satellite sensors can be generally grouped into optical and radar systems. Both systems collect data routinely and at least at moderate resolution, data are often freely available (at the global scale). The quality of global products derived from those sensors depends upon many factors (e.g cloud cover, solar angle, wavelength, etc.), the need for time series, and the availability of ancillary data for validation. A wide spectrum of bands and radiometric resolution offer high information content. However, there is still a limited ability to develop accurate biomass estimation models for tropical forests based on remotely sensed optical data [29]. Early saturation of the signal is the limiting factor in optical systems, along with persistent cloud cover in many of the regions of high biomass over the globe, in particular in the tropical zone. Global optical sensors often process composite images using data covering two to four weeks to avoid cloud and cloud shadow. Medium and high-resolution sensors usually have more problems to obtain cloud free data. In addition, optical data are sensitive to phenological and surface properties of vegetation.

Random errors in the methods applying optical remote sensing for deforestation detection typically range from a few per cent up to 20%, depending on sampling frequency, sample size and deforestation rates, e.g., [30]. Systematic errors occur due to interpretation of satellite images or inappropriate forest classification algorithms and are assessable through ground observations or by analyzing very high-resolution aircraft or satellite data [5] This type of error might be larger in a wall-to-wall approach because a larger area is included. With medium-resolution imagery, systematic errors of 5–20% are achievable for monitoring changes in forest cover when using only two classes (forest and non-forest; [30,31]). The application of satellite or airborne high resolution optical sensors reduces the time and cost of collecting forest inventory data and results in high accuracy. These flights can be undertaken when there is no cloud cover. Such datasets are excellent ground verification for a deforestation baseline but are expensive and technically demanding [3]. The high resolution yields relatively small areas of coverage (e.g.10,000 ha).

Technologies based on Synthetic Aperture Radar (SAR) backscatter depend on the number of scattering elements seen by the radar wave, as well as on their geometric and dielectric properties. These features are directly related to parameters expressing forest density such as the forest growing stock volume [32]. A method for estimating forest biomass maps relates observed backscattering or interferometric coherence data to ground measurements of forest biomass. Improvements in the estimation can be achieved by combining different polarizations and by combining different frequencies. Compared to optical sensors in terms of biomass detection, SAR sensors have the advantage of being able to penetrate clouds and to a limited extent, the forest canopy (dependent upon wavelength). For young and sparse forests the technique achieves high accuracies. The method is less accurate in complex canopies of mature forests because the signal saturates. This can however be compensated somewhat with increasing wavelength and repeated observations. Typically the saturation effect is observed for satellite based SAR systems with low frequencies (P-band) at ~150 tons/ha, and higher frequencies (L-band) at ~100 tons/ha [33,34]. Additionally, in mountainous terrain, the error increases [3].

There are a number of new and innovative technologies which have recently approached operational feasibility, such as light detection and ranging (LiDAR, [28]). LiDAR techniques involve large amounts of data handling and require extensive field data for calibration, which create both a financial and time burden. Airplane-mounted sensors accurately estimate the full spatial variability of forest carbon stocks. This offers a large potential for satellite-based systems to estimate global forest carbon stocks. Future satellite LiDAR systems cannot feasibly "image" from space, but will augment global observations of canopy profiles to more accurately derive biomass at the plot scale. LiDAR systems with large footprints (> 5 m) produce estimates of mean tree height, canopy cover, or canopy density for an area. Regression models constructed using ground measurements, LiDAR data, and ancillary data may then be used to predict accurate estimates of forest carbon stocks [35-37]. Although predictions of diameter and volume may have considerable uncertainty for individual trees, estimates at stand- or plot-level may still be acceptably precise [35].

A 'hierarchical nested approach' combines high and coarse resolution optical, SAR, and/or LiDAR data [38-41]. Coarse resolution optical or SAR is used to identify areas of rapid land use change that then become the focus of further study with higher resolution imagery. This process of sub-sampling can be automated or based on knowledge of deforestation fronts by experts who identify areas of deforestation pressure [42]. A probability-based sampling approach was applied by Hansen et al. [4] that employs MODIS data to identify areas of likely forest cover loss and to stratify probability of forest clearing. Random samples in the strata were interpreted for forest cover and forest clearing by using high-spatial-resolution Landsat imagery. Other data such as maps of infrastructure, population changes in rural areas and maps of policy programs can be used to identify such hot spot areas where a more detailed analysis is required [17]. With the help of such an approach, monitoring systems at national levels in developing countries can also benefit from pan-tropical and regional observations, mainly by identifying hot spots of change and prioritizing areas for monitoring at finer spatial scales [5]. However, finding an adequate sampling method that is dense enough and well designed to capture deforestation events (that are not randomly distributed in space but e.g. along roads, etc.) remains a challenge and will depend on accuracy and precision requirements from the policy process.

Traditional (national) forest inventories (NFI) provide data of the growing stock timber volume per unit area by tree diameter or age classes and species composition. To estimate changes in growing stocks, repeated measurements at permanent sample plots are carried out. There are a few developing countries like India and China that are conducting a national forest inventory on a regular basis [17]. The biomass stock of forest trees in NFIs is usually calculated by using Biomass Expansion Factors (BEFs) that convert timber volumes to dry weight (density factor) and dry weight to whole tree biomass (expansion factor). BEFs are either constant or a function of stand development and exist for many species of temperate forest, e.g. [43,44]. However, there are only a few biomass functions for tropical species [45,46]. Additional destructive biomass measurements would be needed to develop biomass expansion factors and estimate carbon densities. Including such labor intensive activities in an estimation of monitoring costs would of course mean a cost shift at the project level. However, such costs will diminish over time and their reduction can also be achieved by collaboration and scientific exchange.

In many developing countries there are obvious limitations in the availability of appropriate reference data especially for the period 1990–2000. If no robust reference data are available, at a minimum, a consistency assessment should allow some estimation of the forest change quality, i.e. reinterpretation of small samples in an independent manner by regional experts. When completeness or accuracy of estimates cannot be achieved, high uncertainties in input data can be overcome by applying the conservativeness principle [2], which guarantees that the reduction of emissions is not overestimated, or at least the risk of overestimation is minimized. In the context of total emissions from deforestation and degradation, Schlamadinger et al. [47] proposed a corridor to reflect the uncertainty of future emissions. This corridor could be derived using historical emissions, emission trends, and trends in underlying causes.

### Costs of REDD monitoring technologies

Table 2 lists costs for REDD monitoring including remote sensing technologies and inventories. In general, the costs for monitoring will depend on the requirements within REDD which is mainly determined by the accuracy level. The accuracy level will determine the monitoring technology applied, each requiring different ways of data acquisition, processing, training and capacity building. The factors that influence the price of the data acquisition are the amount of ground-based and EO data needed.

**Table 2:** Present acquisition and analysis costs* of monitoring services of various technologies in US$.

**Satellite and sensor**	**Resolution and coverage or project area**	**Costs for data acquisition**	**Cost for analysis**	**Total monitoring costs**	**Source**
**Optical, medium resolution sensors**					
Landsat-5, TM	30 m, 180 × 180 km	0.02 US$/km^2 ^– free	Classification 0.12–0.31 US$/km^2^ Change detection 0.4–0.6 US$/km^2^	0.50–1.21 US$/km^2^	SARMAP pers. comm.
Landsat-7, ETM+	30 m, 60 × 180 km	0.06 US$/km^2^			
SPOT 4	20 m	0.31 US$/km^2^			
Terra ASTER	15 m, 60 × 60 km	0.02 US$/km^2^			
CBERS-2, HRCCD	20 m	free in Brazil			
DMC	32 m, 160 × 660 km	0.04 US$/km^2^			
IRS-P6-LISS III	23.5 m	0.07 US$/km^2^	Human resources and equipment 0.5 US$/km^2^	0.57 US$/km^2^	[19]

**Optical, high resolution sensors**					
Quickbird	3 m	25 US$/km^2^	Classification 2.2–2.5 US$/km^2^ Change detection 4.7–7.9 US$/km^2^	7.50 – 35.40 US$/km^2^	SARMAP pers. comm.
Ikonos	4 m	25 US$/km^2^			
RapidEye	5 m	2.8 US$/km^2^			RapidEye pers. comm.
SPOT-5, HRVIR	5–20 m, 60 × 60 km	0.6 US$/km^2^			SARMAP pers. comm.

**Optical, very high resolution sensors**					
Quickbird	0.6 m	16–22 US$/km^2^	Classification 100–125 US$/km^2^	116–272 US$/km^2^	SARMAP pers. comm.
WorldView-1	0.5 m	16–22 US$/km^2^	Change detection 160–250 US$/km^2^	116–272 US$/km^2^	SARMAP pers. comm.

**Radar, SAR**					
ALOS PALSAR	10–15 m	0.04 US$/km^2^	Classification 2.2–2.5 US$/km^2^	6.94 – 10.44 US$/km^2^	SARMAP pers. comm.
Satellite or shuttle SAR		0.14 US$/km^2^	Change detection 4.7–7.9 US$/km^2^	7.04 – 10.54 US$/km^2^	[60]
Airborne SAR		345 US$/km^2^		> 345 US$/km^2^	[60]

**LiDAR, airborne**					
UK, forest monitoring, national average	28,000 km^2^			415 US$/km^2^	[60]
US, forest inventory at project level	40 km^2^			455 US$/km^2^	[61]
	400 km^2^			100 US$/km^2^	[61]
US, project area	180 km^2^			388 US$/km^2^	[62]
Indonesia, forest inventory at project level	136 km^2^	400–550 US$/km^2^	160 hours processing time	> 400–550 US$/km^2^	RSS GmbH pers. comm.

**Ground-based inventories and national/project examples**					
US, project example	180 km^2^, 1000 sample plots			167 US$/km^2^	[62]
UK, ground survey	28,000 km^2^			172 US$/km^2^	[60]
Bolivia, Noel Kempff Project, inventory	6,340 km^2^; 625 sample plots	17 – 0.16 US$/km^2^**		55 US$/km^2^	[63]
Costa Rica, Private Forestry Project, monitoring	570 km^2^			100 US$/km^2^	[63]
Indian National Forest Inventory and additional biomass assessment	677,088 km^2^; ca. 7,000 NFI plots + 1,400 additional plots			< 10 US$/km^2^	[19]
National Forest Monitoring and Assessment	Total forest monitoring costs of five examples (Zambia, Honduras, Nicaragua, Bangladesh, Cameroon)			1.2 – 8.2 US$/km^2^	[64]
Indonesia, Ulu Masen Project	7,500 km^2^				
RS monitoring and management				81 US$/km^2^	[63]
Airborne monitoring (ultra light aircraft)				200 US$/km^2^	

* Costs for analysis and total costs are indicative costs. They include service design, data processing and mapping, interpretation and analysis. The actual costs would depend on the selected sensor, the fit of sensor data to area to be mapped (which determines how many scenes are needed), the amount of GIS (Geographical Information System) processing, integration and support services required to develop final images and maps and integrate these into asset operational and management systems.

** Variable costs dropped rapidly from a precision level of ± 5 percent to a level of ± 30 percent.

Financial resources for remote sensing assessments of deforestation and degradation are required to acquire suitable satellite data, for processing hardware and software, training and capacity building, data processing and analysis, field work and travel, and for accuracy assessment. The costs for EO are determined by the data quality, resolution, cloud cover, order size, imaging window, and provider. Data processing costs occur for: hardware, software and data analysis. Overall costs depend on factors like existing capabilities and capacities and the comprehensiveness of the monitoring systems. However the costs for data analysis requires special REDD adaptations that depend on the degree of automated processes, effort, and accuracy. Costs that occur in acquiring ground-based data are for field work and travel. Whichever REDD system is applied, it is likely that training and capacity building will be needed in all areas which are part of a monitoring process and there will be costs associated with it.

As stated above, the costs of monitoring REDD are a function of the desired level of precision – which may vary by the size of the project, terrain and heterogeneity of the landscape, location of areas and degree of coherence, and natural variation of carbon pools under observation [6], and also by the required standards of the adopted REDD scheme. Where technical capabilities and cost constraints prevent automated digital analysis, pure manual interpretation of aerial photographs or satellite images is an appropriate monitoring method. The need for reproducible and verifiable results can be met through multiple interpreters and well-designed procedures. For countries with sophisticated data acquisition and analysis, more automated analysis with computer algorithms reduces the time required for monitoring and strengthens the efficiency of the monitoring system in the long term [17].

A transparent form of validation could be achieved by publishing interpreted maps on the internet, or even by allowing public validation of land cover interpretation and land cover change (e.g. [48]).

Building on and enhancing traditional and local level forest governance capacities and establishing community-based forest management systems can be an essential step to efficiently prevent and/or monitor deforestation and degradation [49]. This kind of small-scale forest monitoring is cost-effective, and should bring many more benefits to local communities than other large scale measures, thus contributing more strongly to sustainable development [50]. Research projects reviewed by Skutsch et al. [50] showed that carbon measurement and monitoring methods, which were carried out by community members using hand-held computers with GIS capability and GPS, could accurately map forest resource and carbon stocks at relatively low transaction costs. An efficient policy framework for more community-based management will necessarily involve multi-level governance and involve international, national and local level bodies in developed and developing countries [49].

## Discussion

Remote sensing has been identified as a key technology to successfully implement and monitor a future REDD mechanism [7]. As described above, technological options are manifold. They range from the analysis of coarse resolution data from optical sensors and the application of average values to sophisticated methods of LiDAR and SAR scanning paired with detailed models. All involve different costs and requirements and also yield different accuracies. Finding the optimal technological pathway is crucial for a successful implementation of REDD. The design of a future REDD mechanism has direct implications for the number of monitoring options and also at what costs they can be implemented. But how do monitoring costs relate to other costs of REDD policy implementation and compliance?

### Monitoring costs compared to other costs

Stern [51] identified three types of costs arising from the reduction of deforestation, which are i) opportunity costs previous to the preservation of forests, ii) costs of administration and implementing effective action, and iii) costs of managing the transition. For the purpose of this analysis we differentiate between readiness costs, opportunity costs and implementation costs apart from monitoring costs, the focus of this paper.

### Readiness costs

It is clear at this stage that an important cost component is related to making the participating countries ready for REDD. The vast majority of forest nations participating in REDD will face capacity building costs, i.e. costs for establishing research capacity, technology transfer and legal support. Further costs might include those for land tenure reform and governance reform where these are required to facilitate a REDD financing regime. These so called costs for "readiness" are related to capacity building and policy development to create a framework in which the REDD system can be applied. As these types of costs depend on the national context and do not apply in all cases, they should be regarded as a separate cost type. The Eliasch Review [52] estimates that reforms and capacity building within 40 "forest nations" would cost up to 4 billion US$ over 5 years.

### Implementation costs

Implementation costs of projects in a REDD system depend on the final structure of the REDD mechanism, whether it is market or non market based, implemented at the national or sub national level, etc. They comprise a variety of costs which include: monitoring, planning, verification, certification, enforcement, administration, insurance, brokerage and governance. There will also be costs involved in addressing the risk of leakage in the implementation of a REDD program. In certain cases, this cost could be high (e.g. where degraded land is reforested to provide a substitute forest for sustainable logging, [53]).

As it is unclear what structure a future REDD system will have, cost estimates for the implementation are difficult. However experience has been obtained in specific carbon related projects. The literature values from single projects can only be compared if they indicate what cost items are included and what the framing scheme looks like. Implementation costs typically range from 400 to 1500 US$/km^2^, e.g. [54,55].

When considering these cases for implementation costs for REDD, it has to be taken into account that only specific elements of a future REDD system in local contexts were part of these cases. It is therefore difficult to use these cases for an estimation of implementation costs of a future REDD system. However, it can be seen that each element of the implementation costs has to be considered separately and maybe even looked at in the national/local context to arrive at meaningful estimates.

### Opportunity costs

A key variable for deforestation is how attractive land conversion for individual land owners is, based on the balance between forest value and the value of alternative land-use. Opportunity costs represent the highest alternative land-use of the area under deforestation threat, including net revenue from the conversion itself (e.g. value of extracted timber).

Opportunity costs of REDD have been investigated on a project level. Across Africa, Central America, SE Asia, and South America they amount to 30,000–250,000 US$/km^2 ^([55], see Table 3). There are big uncertainties associated with the estimation of opportunity costs that depend on regional prices and current status of areas that are deforestation candidates. Opportunity costs are particularly sensitive to percentage area harvested and timber price. Subsistence agriculture – a significant cause for deforestation in many tropical regions, has much lower opportunity costs than areas under deforestation threat for commercial agriculture. It has, however, to be considered that the opportunity costs do not necessarily reflect the risk of deforestation. Although low opportunity costs exist, the risk for deforestation may still be high e.g. illegal logging or subsistence agriculture.

**Table 3:** Opportunity costs of avoided deforestation as presented in UNFCCC report Investment and Financial Flows to Address Climate Change [65,66].

**Main/Direct Drivers**	**Area of deforestation/degradation [million ha]**	**Share of total deforested/degraded area [%]**	**Opportunity cost of forest conversion [US$/km^2^]**
Commercial agriculture			
Commercial crops	2.6	20%	224,700
Cattle ranching (large-scale)	1.6	12%	49,800
Subsistence farming			
Small scale agriculture/shifting cultivation	5.5	42%	39,200
Fuel-wood and NTFP* gathering	0.75	6%	26,300
Wood extraction			
Commercial (legal and illegal)	1.8	14%	175,100
Fuel-wood/charcoal (traded)	0.7	5%	12,300
Total	12.9	100%	-

* NTFP are non-timber forest products.

Compared to other cost items, monitoring costs of REDD projects are rather low on a per square kilometre basis. Future monitoring costs are likely to decrease because different sampling intensities will be used building on existing data. Additionally, project implementers will be able to build on previous experience. This is especially true for the more cost intensive monitoring efforts like estimating biomass expansion factors or establishing the first inventory. In particular, opportunity costs rule out expenses that even relatively intensive monitoring would require. As Kindermann et al. [56] estimated through a comparison of global land use models, a 10% reduction in deforestation rates until 2030 would cost about 2,000–25,200 US$/km^2 ^per year as a rent for carbon stocks in forests, assuming a lower (2 US$/t CO_2_) and a higher (10 US$/t CO_2_) value for a global carbon price.

### Co-benefits of integration

There are two types of integration effects with respect to REDD monitoring. The first integration relates to cost savings due to integration of different observation systems/components. When observations are stratified (i.e. higher accuracy and precision in hot spot areas) and different observation systems are integrated to form an observation portfolio (e.g. optical, SAR, LIDAR and in-situ), monitoring costs per MRV REDD unit are minimized. The other integration effect refers to economies of scope when one observing system can yield multiple benefits. In this paper the costs of monitoring for REDD included only the carbon sector. In fact, monitoring of forests has the potential to benefit the development of the forestry sector in general. Those forests monitored for carbon stocks can more easily be assessed for timber supply, the provision of non-timber forest products or other forest functions. Moreover, an integrated monitoring system could result in optimized general land management, including fire management, implementation of land use policies, etc. From an integrated monitoring perspective the currently ongoing discussion on a REDD plus mechanism, that includes measures to lower emissions from deforestation and degradation, also rewards forest management activities that increase carbon stocks in forests. This would help to increase incentives for integrated forest monitoring with associated benefits for the forestry sector.

Other co-benefits exist in the field of ecosystem services with an improved monitoring system, e.g. biodiversity and ecosystem services can be better measured and identified. For example, specific areas of both high carbon value and high ecosystem values can be identified. Additionally, general cadastral mapping services could be supplied by an integrated monitoring system as co-benefits. Disaster management could also be integrated e.g. the Disaster Management Constellation (DMC, ), which is used for many different applications.

However, as previously acknowledged [57], benefits from such an integration can hardly be assessed in detail although their potential has to be considered when monitoring costs are discussed. A good example supporting the value of data integration is the current lack of driver and pressure data for REDD planning. In particular, data from the agricultural sector such as crop maps, information on agricultural management practices, farm size ownership structure and land tenure, all the way to education and health data are virtually impossible to compile from existing statistics. This fact points to yet another dimension of REDD monitoring, which goes far beyond forest carbon accounting. For implementation planning and subsequent monitoring and evaluation of REDD policies and activities, such data will be indispensable. The question of how such data should be compiled will have a crucial impact on costs. A classical top-down approach of governmental agencies collecting data will likely not be feasible if REDD is to be implemented through local projects. Bottom-up citizen sensor approaches and web-based information from third party audits might lead (in such a scenario) to information disclosure. On the contrary, if REDD is to be implemented through national REDD programs, the existing statistical apparatus might be sufficient to handle additional REDD monitoring requirements.

### REDD monitoring as part of GEO

Global initiatives e.g. the Group on Earth Observations (GEO), claim that better international cooperation in the collection, interpretation, and sharing of EO information is an important and cost-effective mechanism for improving information available to decision makers. Fritz et al. [57] asked how the benefits of EO can be assessed to justify the additional investment required to facilitate international collaboration, data sharing, linking the current observing systems and to reach interoperability among the current observing systems. They proposed a "benefit chain" concept, based on the logic that an incremental improvement in the observing system (including its data collection, interpretation and information-sharing aspects) will result in an improvement in the quality of decisions based on that information.

In this paper we assess costs of REDD monitoring. These include information that are available from implemented or planned projects and monitoring companies (the latter to a smaller degree). In the case that information from REDD projects could be fed into GEO and contribute to the Global Earth Observation System of Systems (GEOSS), benefits for society could be manifold. Therefore, some of the REDD costs could be offset by benefits to the forestry sector and potentially to many other of the societal benefit areas such as water, health, energy, disaster, biodiversity, ecosystems, climate and weather.

Applied to the case of REDD policy and monitoring requirements, the benefit chain in REDD policy with respect to a better observing system could be described as follows: improved accuracy of monitoring of forest carbon stock changes leads to a better constraint on potential emissions from these forests and more realistic baselines, therefore giving the REDD process much more credibility overall, leading in the end to lower insurance costs.

One resulting question from the GEOSS perspective is how costs are going to be distributed globally in a REDD framework. An appropriate answer would probably require at least a general assessment of the potential distribution of the benefits. This will remain a major challenge.

## Conclusions

The design of a REDD policy framework (specifically its rules), can have a substantial effect on monitoring costs. Nevertheless, many of the technical challenges of monitoring emissions from deforestation (and to a lesser extent degradation) are feasible. Moreover, costs of REDD monitoring are affordable and relatively low compared to other cost items that occur in REDD (often below 10% of total costs).

Future monitoring costs are likely to decrease because different sampling intensities will be used, project implementers can build on previous experience and existing data, and advances in technology will be available. If the advantages of co-benefits in other sectors (optimized land management, improved fire management, agricultural monitoring, etc.) are included in a cost benefit analysis, costs of REDD monitoring will further decrease. Considering REDD as part of a Global Earth Observation System of Systems (GEOSS) will help to realize these benefits.

International cooperation is, however, needed to overcome initialization costs, unequal access to monitoring technologies and know-how.

## Competing interests

The authors declare that they have no competing interests.

## Authors' contributions

HB coordinated the work on this paper, designed structure and concept and is the major contributor to the text. KE provided data and substantially contributed to several paragraphs of the manuscript. GK helped with critical revision and literature input. SF and FK incorporated the links to the GEO process. IM provided literature and data and helped drafting paragraphs of this paper. MO had the idea, contributed to the design of the study and critically reviewed the manuscript. All authors read and approved the final version of the manuscript.
